# Rational Use of Mechanical Circulatory Support as a Bridge to Pediatric and Congenital Heart Transplantation

**DOI:** 10.21470/1678-9741-2018-0081

**Published:** 2018

**Authors:** Leonardo A. Miana, Guilherme Viotto Rodrigues da Silva, Luiz Fernando Caneo, Aida Luisa Turquetto, Carla Tanamati, Gustavo Foronda, Maria Raquel Massoti, Juliano G. Penha, Estela Azeka, Filomena R. B. G. Galas, Fabio B. Jatene, Marcelo B. Jatene

**Affiliations:** 1 Cardiovascular Surgery Division, Instituto do Coração do Hospital das Clínicas da Faculdade de Medicina da Universidade de São Paulo (InCor-HCFMUSP), São Paulo, SP, Brazil.

**Keywords:** Heart Transplantation, Heart Defects, Congenital, Heart-Assist Devices, Heart Failure/Therapy

## Abstract

**Introduction:**

Donor shortage and organ allocation is the main problem in pediatric heart transplant. Mechanical circulatory support is known to increase waiting list survival, but it is not routinely used in pediatric programs in Latin America.

**Methods:**

All patients listed for heart transplant and supported by a mechanical circulatory support between January 2012 and March 2016 were included in this retrospective single-center study. The endpoints were mechanical circulatory support time, complications, heart transplant survival and discharge from the hospital.

**Results:**

Twenty-nine patients from our waiting list were assessed. Twelve (45%) patients were initially supported by extracorporeal membrane oxygenation (ECMO) and a centrifugal pump was implanted in 17 (55%) patients. Five patients initially supported by ECMO were bridged to another device. One was bridged to a centrifugal pump and four were bridged to Berlin Heart Excor®. Among the 29 supported patients, 18 (62%) managed to have a heart transplant. Thirty-day survival period after heart transplant was 56% (10 patients). Median support duration was 12 days (interquartile range [IQR] 4 - 26 days) per run and the waiting time for heart transplant was 9.5 days (IQR 2.5-25 days). Acute kidney injury was identified as a mortality predictor (OR=22.6 [CI=1.04-494.6]; *P*=0.04).

**Conclusion:**

Mechanical circulatory support was able to bridge most INTERMACS 1 and 2 pediatric patients to transplant with an acceptable complication rate. Acute renal failure increased mortality after mechanical circulatory support in our experience.

**Table:** 

Abbreviations, acronyms & symbols				
AKI	= Acute kidney injury		CS	= Mechanical circulatory support
BNP	= Brain natriuretic peptide		MODs	= Multiple organs dysfunction
CHD	= Congenital heart disease		pRIFLE	= Pediatric risk, injury, failure, loss, end stage renal disease
CPB	= Cardiopulmonary bypass		RV	= Right ventricle; right ventricular
CT	= Computed tomography		SIRS	= Systemic inflammatory response syndrome
ECMO	= Extracorporeal membrane oxygenation		TEE	= Transesophageal echocardiography
HTx	= Heart transplantation		UFH	= Unfractionated heparin
IQR	= Interquartile range		USA	= United States of America
LV	= Left ventricle		VAD	= Ventricular assist device
MCD	= Massive cerebral damages			

## INTRODUCTION

Heart failure is a complex pathophysiological syndrome that can occur in children due to a variety of diseases, including cardiomyopathies, myocarditis, and congenital heart disease (CHD)^[^^1^^]^. The incidence of dilated cardiomyopathy from large population-based studies in the United States and Australia range from 0.57 to 0.73 per 100000 children per year^[^^2^^,^^3^^]^. Among these patients, only 66% were alive without heart transplant one year after diagnosis^[^^4^^]^.

Heart transplantation (HTx) is increasingly accepted as the gold-standard treatment for end stage heart disease (either functional, anatomic or both) in the pediatric and adult congenital population^[^^5^^,^^6^^]^. Donor shortage and difficulties in allocation increase mortality during the waiting list for transplantation, specifically in low-weight receptors, and high-risk congenital heart and cardiomyopathy patients that develop cardiogenic shock^[^^7^^]^.

The use of mechanical circulatory support (MCS) systems as a bridge to transplantation or bridge to recovery are the main indications in infants and children^[^^8^^-^^11^^]^. Recently published data from the International Society for Heart and Lung Transplantation reported that approximately 25% of pediatric patients receive some kind of MCS before HTx^[^^12^^]^. Long and short-term MCS is associated with higher survival to HTx in these patients^[^^13^^]^.

Although the majority of Latin America countries faces serious economic issues, in order to increase survival in advanced heart failure, the implementation of MCS programs is welcome in these locations.

The waiting list time in Latin America is three times higher than in United States of America (USA) and two times higher than in Europe^[^^8^^]^. Mean waiting list time for transplantation in Brazil is 6 months, but in patients less than 5 kg this period is greater than 10 months^[^^8^^]^.

HTx program in Brazil is financed by the government and until now, there is no financial support for any kind of MCS. Nevertheless, a small percentage of patients are insurance covered that may have access to MCS technology and few Brazilian institutions support an MCS program financed by research grants.

Our institution represents the largest Pediatric HTx Program in Latin America. We have performed an average of 17 pediatric and congenital HTx annually in the last five years, and our waiting list mortality, especially for the patients classified as INTERMACS 1 and 2, is higher than 80%^[^^7^^,^^13^^]^. Our MCS program is supported by our institutional fund and restricted to extracorporeal membrane oxygenation (ECMO) and centrifugal pumps (Rotaflow® - Maquet Getting Group, Rasttat, Germany).

The present study aims to evaluate initial results, risk factors, lessons learned and future directions after this experience.

These are the first series of a mechanically supported bridge to transplant pediatric and congenital heart patients including short and long-term support devices published in Latin America.

## METHODS

A retrospective study using electronic medical record of 29 consecutive patients listed to HTx who required MCS between January 2012 and March 2016 at our institution.

The institutional ethics committee on human research under the number CAAE 60236316.5.0000.0068 approved this study and due to the retrospective nature of the study, the need for individual patient consent was waived.

### Timing and Indications

ECMO indications as a bridge to HTx or bridge to bridge was mainly in cardiogenic shock or post-cardiotomy cardiac failure (INTERMACS level 1). Neck vessels assessment was the elected cannulation site, but central cannulation was approached when there was a recent previous sternotomy or in post-cardiotomy patients.

Centrifugal pumps (Rotaflow® - Maquet Getting Group, Rasttat, Germany, and PediVas® or Centrimag® - Thoratec Corporation, USA) were implanted mainly in waiting list patients with rapid clinical deterioration despite inotropic support (INTERMACS 2 level) or in two INTERMACS level 1 cases where an ECMO circuit was not available at the time. Patients were cannulated using bypass cannulas or Berlin Heart EXCOR® cannulas. The preferred cannulation sites were left ventricle (LV) apex and aorta, but in the beginning of the cohort and in restrictive cardiomyopathy cases, left atrium cannulation was preferred. When LV cannulation was used, the procedure was performed under cardiopulmonary bypass (CPB) and beating heart. When biventricular support was indicated, right atrium and pulmonary artery were cannulated.

Right ventricular (RV) support was considered in the presence of severe RV dysfunction or in case of moderate RV dysfunction with a central venous pressure above 15 mmHg and persistently depressed RV function after LV decompression guided by transesophageal echocardiography (TEE) at the operating room.

Berlin Heart Excor® was implanted in four cases, always after an ECMO run for circulatory resuscitation. The implant was performed under CPB and beating heart with LV apical and aortic cannulae.

### Management

Device selection was performed based on the initial clinical status, where INTERMACS level 1 was a primary indication for ECMO and INTERMACS level 2 was an indication for a centrifugal pump. Due to our financial restriction, INTERMACS level 3 was not an indication for MCS in our institution. In cases where ECMO was the first-line therapy, conversion to centrifugal pump or Berlin Heart Excor® was attempted after 5 to 10 days of ECMO initiation and clinical stabilization. In two cases where an ECMO circuit was not available, MCS was achieved using a centrifugal pump.

Conversion to a centrifugal pump was considered in one case in the context of the potential for recovery (post-cardiotomy ECMO in a single ventricle physiology) to minimize complications while performing a thorough transplant assessment (*e.g*., neurologic assessment) and because renal replacement therapy or respiratory support was not necessary at the time.

Conversion from a centrifugal pump to an ECMO circuit occurred in one patient who developed multi-organ failure during centrifugal pump run and needed renal replacement therapy and prolonged respiratory support.

Anti-coagulation was based on the Edmonton protocol^[^^14^^]^ where unfractionated heparin (UFH) was started within 12 to 24 hours of device implantation, depending on the degree of postoperative bleeding based on chest tube output. An acceptable chest tube output as a trigger for starting UFH was considered if less than 2 ml/kg/h. UFH was titrated to a target anti-Xa level of 0.35 to 0.6 U/ml, with a goal activated clotting time range that correlated with the anti-Xa target. Anti-coagulation goals and agents were adjusted individually depending on the circuit condition as well as on the patient's bleeding and thrombotic profile.

### Outcomes

The primary outcome of this study was decannulation from any type of MCS due to transplantation, recovery or death. Complete follow-up data were available for all patients.

Five types of MCS-related complications were accessed in this cohort and included bleeding, neurologic events, infection, ischemic organ damage, and mechanical device failure.

Bleeding as the requirement for re-exploration for bleeding or hematoma were considered significant.

Neurologic events included intracranial bleeding or ischemic stroke diagnosed by computed tomography (CT) scan.

Infections were defined as the presence of positive culture from blood, urine, intravenous catheter, or sputum with associated clinical symptoms. In the absence of a defined positive culture, antibiotic use alone was not considered as an evidence of infection, but clinical symptoms with suggestive laboratory findings.

Acute kidney injury (AKI) in any time of treatment was defined according to pediatric Risk, Injury, Failure, Loss, End Stage Renal Disease (pRIFLE) score^[^^15^^]^.

Finally, mechanical device failure was defined as a malfunction of one or more components rendering the system incapable of functioning and requiring a device exchange. This did not include exchanges for pump thrombosis or clots in the circuit, considered as thrombotic events.

Circuit thrombosis included any identified cloth in the circuit with or without a need for pump, membrane or complete circuit exchange.

All data were analyzed to identify risk factor for survival to transplantation, survival to hospital discharge and stroke.

Studied risk factors included age, weight, gender, primary diagnosis (cardiomyopathies x CHD), univentricular heart physiology, type of primary MCS (ECMO x centrifugal pump), number of MCS implants, preoperative cardiac arrest, preoperative AKI, AKI during hospitalization, stroke, preoperative mechanical ventilation, systemic inflammatory response syndrome (SIRS), preoperative serum creatinine value, preoperative serum total bilirubin value, preoperative serum lactate value, preoperative serum brain natriuretic peptide (BNP) value, infection, major bleeding and circuit thrombosis.

The time of support was considered as the time between the implantation and any outcome (transplantation, explant or death).

### Statistical Analysis

Descriptive statistics were presented in the median with interquartile range (IQR) for continuous variables and number and percentile for the other variables. Normality test used was Shapiro-Wilk. Chi square, Mann-Whitney, and Exact Fisher test were used to compare groups. Binary logistic regression with multivariable analysis and Hosmer-Lemeshow goodness of fit test was used to identify risk factors for mortality and stroke. A *P* value < 0.05 was considered significant. The software SPSS 21.0 (SPSS, Chigago, IL, USA) was used for statistical analysis.

## RESULTS

Twenty-nine patients listed for HTx had at least one MCS implanted. Twelve patients in refractory cardiogenic shock or postcardiotomy cardiac failure (INTERMACS level 1) were supported initially by an ECMO circuit. The other 17 patients, mostly classified as INTERMACS level 2 underwent centrifugal pump implantation.

Demographic and preoperative variables are listed in Table 1.

**Table 1:** Pediatric and congenital heart transplant patients receiving mechanical circulatory support demographics comparing centrifugal pump and ECMO patients.

Variables	All patients (n=29)	Centrifugal Pump (n=17)	ECMO (n=12)	*P* value
Age at implant (years), mean (IQR)	5.6 (1.8-12)	5.2 (1.8-9.5)	8.8 (1.5-19.4)	0.2
Weight at implant (kg), mean (IQR)	16.7 (8.9-35)	16 (10.5-29)	24.5 (7.1-52.5)	0.5
Male sex, N (%)	15 (51.7%)	8 (47%)	7 (58%)	0.7
Number of MCS implant				
1	23	16	7	0.06
2	6	1	5	
Diagnosis at implant				
Cardiomyopathy/myocarditis	21 (72.4%)	15 (88.2%)	6 (50%)	0.04
Congenital heart disease	8 (27.6%)	2 (11.8%)	6 (50%)	
MCS in postoperative cardiac surgery	6 (20.7%)	1 (5.9%)	5 (41.7%)	0.06
Pre-MCS mechanical ventilation	20 (69%)	12 (70.6%)	8 (66.7%)	0.9
Pre-MCS cardiac arrest, N (%)	16 (55.2%)	11 (64.7%)	5 (41.7%)	0.3
Pre-MCS AKI, N (%)	12 (41.4%)	6 (35.3%)	6 (50%)	0.5
Pre-MCS creatinine plasma levels, average (STD)	0.7 (0.4-1.06)	0.6 (0.4-0.8)	1 (0.4-1.4)	0.4
Pre-MCS total bilirubin plasma levels, average (STD)	1 (1-1.5)	1 (1-2)	1 (0.7-1.3)	0.3
Pre-MCS arterial lactate plasma levels, average (STD)	16 (13-26.5)	17 (13.5-25)	13.5 (13-26.7)	0.4
Pre-MCS BNP plasma levels, average (STD)	2782 (1170-4466)	3207 (1560-4475)	1170 (454-4292)	0.2

AKI=acute kidney injury; BNP=brain natriuretic peptide, ECMO=extracorporeal membrane oxygenation; HTx=heart transplantation; IQR=interquartile range; MCS=mechanical circulatory support; STD=standard deviation

**Fig. 1:**
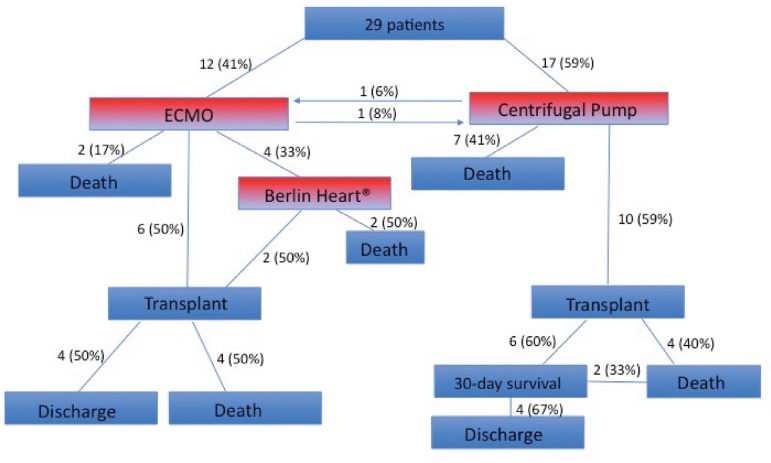
Outcomes in INTERMACS level 1 and 2 Pediatric and Congenital Heart Transplant waiting list patients connected to MCS.

Five (41.7%) ECMO patients were bridged to another device before the HTx. One of them received a centrifugal pump and the other four were connected to a paracorporeal ventricular assist device (VAD; Berlin Heart Excor®). Two (16.7%) ECMO patients died during MCS run. One patient died due to multiple organ failure and a massive circuit thrombosis was found in the other patient leading to death. Six (50%) patients received an HTx during ECMO run (Figure 1).

Among the four Berlin Heart patients, two (50%) were transplanted and two died during MCS, one due to sepsis and one due to multiple organs dysfunction (MODs).

Seven (41.2%) centrifugal pump patients died during MCS run, five of them due to MODs and two due to massive cerebral damages (MCD). While 58.8% (10 patients) managed to have a HTx (Figure 1).

Median time in MCS was 12.4 days (IQR= 4.3-26.3 days) and there was no correlation between time on support and survival. There was no difference in time of support or any other result variable between initial types of support (Table 2). HTx was achieved in 18 (62%) patients with 55.5% (10 patients) 30-day survival, while eight (44.5%) patients survived to discharge.

**Table 2:** Pediatric and congenital heart transplant patients receiving mechanical circulatory support results comparing centrifugal pump and ECMO patients.

Variables	All patients (n=29)	Centrifugal Pump (n=17)	ECMO (n=12)	*P* value
Duration of support (days), mean (IQR)	12.4 (4.3-26.3)	12.4 (3-25)	13.3 (6-45)	0.6
Infective complication, n (%)	13 (44.8%)	6 (35.3%)	7 (58.3%)	0.3
Circuit thrombus formation, n (%)	7 (24.1%)	3 (17.6%)	4 (33.3%)	0.4
Chest reexploration, n (%)	7 (24.1%)	3 (17.6%)	4 (33.3%)	0.2
AKI during hospitalization, n (%)	15 (51.7%)	8 (47%)	7 (58.3%)	0.9
Cerebral injury - n (%)	17 (58.6%)	11 (64.7%)	6 (50%)	0.5
Survival to Htx - n (%)	16 (55.2%)	10 (58.8%)	6 (50%)	0.7
30-day Survival - n (%)	10 (62.5%)	6 (60%)	4 (66.7%)	0.7

AKI=acute kidney injury; BNP=brain natriuretic peptide; ECMO=extracorporeal membrane oxygenation; HTx=heart transplantation; IQR=Interquartile range; MCS=mechanical circulatory support; STD=standard deviation

### Comparisons

All causes of post HTx death and patients characteristics are listed in Table 3.

**Table 3:** Causes of death after heart transplant.

Patient	Diagnosis	MCS	Duration of MCS (hours)	Cause of Death	Comments
1	MCP	CP	654	MCD	__
2	CHD (TOF)	CP	34	MCD	__
3	MCP	CP	79	Sepsis	__
4	CHD (SV)	CP	297	MOD	__
5	MCP	CP	44	Sepsis	Biventricular CP; ECPR 21 days post-HTx
6	MCP	ECMO+BH	ECMO bridge to bridge: 480 hours BH: 330 hours	MCD	__
7	CHD (SV)	ECMO	123	MCD	__
8	MCP	CP+ECMO	Rotaflow bridge to bridge: 48 ECMO bridge to HTx: 536	MOD	ECPR immediate post-HTx
9	CHD (SV)	ECMO	223	MCD	__
10	CHD (PA/VSD)	ECMO	58	Sepsis	__

BH=Berlin Heart Excor^®^; CP=centrifugal pump; ECMO=extracorporeal membrane oxygenation; HTx=heart transplantation; MCD=massive cerebral damage; MCP=cardiomyopathy; MCS=mechanical cardiac support; MOD=multiple organs disfunction; PA/VSD=pulmonary atresia with ventricular septal defect; SV=single ventricle physiology

Sixteen (55.2%) patients had at least one resuscitated cardiac arrest before MCS implant. Any kind of cerebral damage was identified in 17 (58.6%) patients. On the other hand, circuit thrombosis or preoperative cardiac arrest were not risk factors for cerebral damage (*P*=0.9 and *P*=0.6, respectively).

Seven patients died from MCD, five post-Htx (Table 3) and two during CP run. Six other patients affected by cerebral damage died during MCS run, but the cerebral damage was not the main cause of death in these patients.

Among the eight patients that were discharged home, four presented some kind of cerebral damage. Three of them did not have any sequelae, but one patient suffered significant motor impairment and needed a specialized assistance at home after discharge.

Other complications during MCS run included infection in any site in 13 (44.8%) patients, but none were mediastinitis or cannulae related infection. Respiratory tract (6 cases), blood stream infection (4 cases) and urinary tract (3 cases) were the identified sites of infection.

In seven (24.1%) patients, circuit thrombus was identified and six of them were submitted to circuit replacement. One patient presented sudden massive ECMO circuit thrombosis and circuit change was impossible leading to death. No mechanical device failure was detected, except due to thrombosis.

Six (20.1%) patients presented increased mediastinal bleeding and need for chest re-exploration. AKI was observed in 16 (55.2%) patients and among them, 12 (75%) had already presented AKI before MCS implantation. Mortality in AKI patients was 94% compared to 46% in preserved renal function cases (*P*=0.01).

It was not observed any case of significant ventricular function recovery leading to MCS discontinuation. On the other hand, 11 patients died while on MCS, seven due to MODs, two due to massive cerebral stroke, one due to sepsis and one due to circuit thrombosis. Univariate analysis showed AKI during hospitalization and major bleeding as a risk factor while multivariate analysis identified AKI as a predictor of death during support (OR=50.8; IC: 1.9-1370; *P*=0.02).

Regarding survival to hospital discharge, univariate analysis showed BNP over 1000 post-MCS, SIRS, and AKI as risk factors, but multivariable analysis and Binary logistic regression identified that only AKI during hospitalization was a predictive variable of mortality (OR=22.6 [CI=1.04-494.6]; *P*=0.04; Table 4).

**Table 4:** Univariate and multivariate analysis of death.

Variables	Univariate OR (CI)	Univariate *P* value	Multivariate OR (CI)	Multivariate *P *value
BNP post-MCS	3.1 (1.1-62)	0.04	3.2 (0.1-81)	0.5
SIRS	9.5 (1.3-71)	0.03	9.6 (0.3-300)	0.2
AKI	17.5 (1.8-175)	0.01	22.6 (1.04-494)	0.047

AKI=acute kidney injury; BNP=brain natriuretic peptide; CI=confidence interval; MCS=mechanical cardiac support; OR=odds ratio; STD=standard deviation

## DISCUSSION

Pediatric and congenital HTx programs have an intrinsic complexity that mixes elective dilated cardiomyopathy patients with cardiogenic shock and complicated congenital hearts. Besides that, waiting time on list tends to be a lot longer than that for bigger patients, due to the donor scarcity^[^^8^^,^^16^^]^.

Definitely, heart failure pediatric patients that present with a critically ill condition have a bad prognosis, especially in our environment. Our group previously reported less than 10% on 30-day survival in non-supported cardiogenic shock cohort^[^^7^^]^.

The International Society for Heart and Lung Transplantation report demonstrates that the number of pediatric HTx previously supported by MCS grows exponentially every year^[^^8^^]^.

Although facing a serious public health financing crisis in our country, especially concerning new technology, institutional politics stated that MCS should be implemented in our HTx program. Counting almost exclusively with ECMO circuits and centrifugal pumps (Rotaflow®, Maquet Getting Group, Rasttat, Germany) was possible to offer an alternative to the proven failed conventional treatment to these patients.

It was necessary a lot of commitment and investment in staff training leading to improving results with MCS previously demonstrated in a series of post-cardiotomy ECMO^[^^17^^]^. Paracorporeal VAD (Berlin Heart Excor®) and Centrimag (Thoratec Inc, USA) were available only for insurance covered patients that represent the vast minority of our patients.

As short-term MCS, ECMO and centrifugal pump were used, while Berlin Heart Excor® was the only long-term device implanted in this initial experience, available in selected insurance covered patients.

Our initial results showed an improvement in survival to HTx (55.2%) in these patients compared to our historical cohort where a 10% survival to HTx was observed^[^^7^^]^. Although these results cannot be compared to developed countries' experience^[^^18^^,^^19^^]^, it may be considered the first step towards a successful program.

Concerning that, during the initial experience with Berlin Heart for pediatric patients, the results were very unsatisfactory with a 100% mortality for patients aged less than one year^[^^20^^]^. Therefore, after the learning curve and program adjustments in cannulation and avoidance of late indications, their results improved dramatically^[^^20^^,^^21^^]^.

Long-term MCS is not routinely available for government or insurance funded patients. This scenario imposes late indications for MCS as a bridge to HTx, mainly in patients in critically-ill condition (INTERMACS levels 1 and 2) and using short-term MCS what might, in part, explain suboptimal results. Indisputably, most of these issues were caused by the lack of long-term support, leading to long courses of short-term MCS and its well-known complications. Even Berlin Heart Excor® patients in our series were supported for a considerable amount of time on ECMO while waiting for bureaucratic issues. That lead to a 50% mortality during Berlin Heart run caused by sepsis and multi-organ failure.

Recent data suggest that patients who underwent ECMO support have inferior post-transplant survival when compared to those who underwent VAD bridge to transplantation or direct transplantation. Patients in ECMO group had a lower median age and were significantly smaller^[^^16^^]^.

Meanwhile, between 2006 and 2011, Great Britain had a fourfold increase in their Berlin Heart use in the pediatric population. A retrospective study of their first 7-year experience in 102 children who received Berlin Heart Excor® support, 84% survived to transplant or explant of the VAD, and 81% survived to discharge^[^^18^^]^.

As the first report from PediMACS, analyzing 200 pediatric patients supported with durable VADs, showed a 1-year survival rate of 81%. Approximately 60% of all patients were transplanted in the 6^th^ month and 75% in the 12^th^ month. On the other hand, survival was significantly lower in patients who were in INTERMACS level 1^[^^19^^]^, what might in part explain our high mortality.

It is well known that there is a considerable gap between excellence medical centers accomplishments and Latin America follow through^[^^22^^]^. Nevertheless, as in Brazil, some isolated efforts in Argentina and Colombia are noticed in this direction leading to promising results, as the recently published Garrahan's group initial experience with MCS^[^^23^^]^.

Even though, a lot of concern was raised regarding our mortality after MCS patients HTx. A 37.5% 30-day mortality rate was much higher than the less than 10% mortality observed in non-MCS supported patients previously reported by our group^[^^24^^]^.

Trying to understand the results we noticed that Htx was considered and even performed in very sick patients supported with MCS. At that time, neglected neurologic damage and underestimated multiple organ dysfunctions drove us to HTx in irreversible non-cardiac malfunctions.

Although a high percentage of SIRS and cerebral damage was identified in this series, AKI was the only identified isolated risk factor for mortality. Nevertheless, it is well known that AKI and MODs are correlated.

That fact led us to improve our protocol and withdraw patients from the waiting list after the initiation of MCS and re-listing them only after better neurologic and multi organic assessment, preferably with spontaneous breathing patients.

Still, new challenges remain regarding the incorporation of MCS by Latin America centers. Apart from financial support, continuous education and training are required to use these devices and is the key point to achieve excellence centres' results. International partnership plays an important role in this scenario, where foreign assistance should be adjusted to the local context avoiding dropping a replica of a proven model into an obsolete system.

Limited resources are a barrier to the development of academic, teaching, and cost-effectiveness MCS programs in Latin America. These programs should be designed based on the local needs, centralized in active transplants centers and financed by the public national systems.

## CONCLUSION

Our initial results showed that MCS was able to bridge most INTERMACS 1/2 pediatric and congenital heart patients to HTx. The poor clinical condition of this population and the lack of largely available long-term MCS could explain the sub-optimal mortality observed in this series, where AKI was identified as a risk factor.

**Table:** 

Authors' roles & responsibilities	
LAM	Concept/design; drafting article; statistics, data interpretation; final approval of the manuscript version to be published
GVRS	Data collection; data interpretation; final approval of the manuscript version to be published
LFC	Concept/design; data interpretation; final approval of the manuscript version to be published
ALT	Statistics; final approval of the manuscript version to be published
CT	Data interpretation; final approval of the manuscript version to be published
GF	Data collection; final approval of the manuscript version to be published
MRM	Data analysis; final approval of the manuscript version to be published
JGP	Data interpretation; final approval of the manuscript version to be published
EA	Concept/design, critical revision of the manuscript; final approval of the manuscript version to be published
FRBGG	Concept/design, critical revision of manuscript; final approval of the manuscript version to be published
FBJ	Final approval of the manuscript version to be published
MBJ	Critical revision of the manuscript; final approval of the version to be published
